# Comparison of Neuroprotective Effect of Bevacizumab and Sildenafil following Induction of Stroke in a Mouse Model

**DOI:** 10.1155/2016/3938523

**Published:** 2016-05-29

**Authors:** Ivan Novitzky, Neelan J. Marianayagam, Shirel Weiss, Orkun Muhsinoglu, Moran Fridman, Tamar Azrad Leibovitch, Nitza Goldenberg-Cohen, Shalom Michowiz

**Affiliations:** ^1^Department of Neurosurgery, Rabin Medical Center-Beilinson Hospital, 49100 Petach Tikva, Israel; ^2^Krieger Eye Research Laboratory, Felsenstein Medical Research Center, 49202 Petach Tikva, Israel; ^3^Sackler Faculty of Medicine, Tel Aviv University, 6997801 Tel Aviv, Israel; ^4^Pediatric Ophthalmology Unit, Schneider Children's Medical Center of Israel, 49100 Petach Tikva, Israel

## Abstract

To evaluate the effect of bevacizumab and sildenafil on stroke parameters in a mouse model, middle cerebral artery occlusion was induced in male C57Bl/6 mice using an intra-arterial filament method. The filament was removed after 60 minutes, and the mice were immediately given a single intraperitoneal injection of saline, bevacizumab, or sildenafil. An additional group of mice (*n* = 7) received bevacizumab 6 h after MCAO induction. The mice were euthanized 24 hours later and evaluated for infarct area and brain edema using triphenyltetrazolium chloride staining and ImageJ. In the saline-treated mice (*n* = 16), total stroke volume was 19.20 ± 6.38 mm^3^, mean penumbra area was 4.5 ± 2.03 mm^3^, and hemispheric asymmetry was 106.5%. Corresponding values in the bevacizumab group (*n* = 19) were 17.79 ± 5.80 mm^3^, 7.3 ± 3.5 mm^3^, and 108.6%; in the delayed (6 h) bevacizumab injected mice (*n* = 7) they were 9.80 ± 8.00 mm^3^, 2.4 ± 2.0 mm^3^, and 98.2%; and in the sildenafil group (*n* = 16) they were 18.42 ± 5.41 mm^3^, 5.7 ± 2.02 mm^3^, and 109.9%. The bevacizumab group had a significantly larger mean penumbra area when given immediately and smaller total stroke area in both groups than the saline- (*p* = 0.03) and sildenafil-treated (*p* = 0.003) groups. Only delayed bevacizumab group had reduced edema. Bevacizumab, injected immediately or delayed after injury, exerts a neuroprotective/salvage effect, whereas immediate treatment with sildenafil does not. Inflammation may play a role in the neuroprotective effect.

## 1. Introduction

Bevacizumab is an inhibitor of vascular endothelial growth factor (VEGF) that targets VEGF-A, an isoform of VEGF that stimulates endothelial cell proliferation and migration. By specifically binding the VEGF-A protein, it reduces the VEGF protein levels and restricts the growth of abnormal blood vessels, thus reducing their leakage. In addition, it has an anti-inflammatory role that reduces macrophage activity and inhibits infiltration of inflammatory materials causing neuronal damage [1]. Bevacizumab has been found to exert an antiangiogenic effect in tumors and a neuroprotective effect in the brain by reducing leakage and edema [1]. It is used in the treatment of colon cancer [2] and gliomas (low and high grade) [3] and age-related macular degeneration [4]. It was also recently found to be effective in reducing macular edema in the sensory retina of patients with diabetes [5]. However, some studies suggest that bevacizumab may play a role in causing cerebral ischemia [6, 7].

Sildenafil citrate is a potent vasodilator which inhibits the action of phosphodiesterase 5 (PDE 5). Sildenafil competes with cyclic guanosine monophosphate (cGMP), responsible for smooth muscle relaxation, for the substrate-binding site on PDE 5, thereby increasing the concentration of cGMP in the tissue [8]. Sildenafil is used mainly in the treatment of erectile dysfunction. It is beneficial for the treatment of pulmonary hypertension as well, as PDE 5 is also distributed in the smooth muscle arterial walls of the lungs [9]. It was also found to induce retinal artery and vein dilatation in healthy subjects [10]. Recent studies have shown that sildenafil may exert a protective effect against ischemic injury in various organ systems such as the heart and kidney [11–13]. In cardiac tissue, it acts as a mimetic for super oxide dismutase, preventing the generation of hydrogen peroxide (H_2_O_2_) [11]. This reduces the formation of oxygen-free radicals, the driving force behind ischemia reperfusion injury and consequent mitochondrial dysfunction and cardiac myocyte injury. Sildenafil also protects the heart by activating the ATP-sensitive potassium channels on the mitochondrial walls [12], thereby helping to maintain the mitochondrial membrane potential during ischemia reperfusion injury. Furthermore, sildenafil has at least a partial anti-inflammatory effect through iNOS inhibition [14]. It was found to reduce the expression of cytokines, COX-2, and GFAP (glial fibrillary acidic protein) in a demyelinating model induced in wild type mice, and in iNOS^−/−^ mice, sildenafil reduced Iba-1, IFN-*γ*, and IL-1*β* levels following cuprizone induced myelin damage, and increased glutathione S-transferase pi, improving the myelin structure/ultrastructure [14]. Prompted by findings that sildenafil citrate is protective against ischemia reperfusion injury in neonatal rats, researchers postulated that it may be helpful in the treatment of neonatal cerebral ischemia [15]. At the same time, several reports have described sildenafil as a stroke inducer in the presence of preexisting conditions or a history of stroke [16].

The aim of the present study was to determine if bevacizumab and sildenafil have a protective influence on cerebral ischemic events using a mouse model of stroke.

## 2. Methods

### 2.1. Animals

All protocols were conducted in accordance with the ARVO Statement for the Use of Animals in Ophthalmic and Visual Research and were approved and monitored by the Animal Care Committee of Rabin Medical Center (Permit Number 030813, 022-b6640-2). Adult male C57Bl/6 mice (wild type) were purchased from Harlan Laboratories (Jerusalem, Israel). Animals were maintained in our animal colony at 22°C on a 12-hour light/12-hour dark cycle. All procedures and analyses were performed by the same researcher (Ivan Novitzky) to reduce variability.

### 2.2. Induction of Middle Cerebral Artery Occlusion

Our middle cerebral artery occlusion (MCAO) model is based on previously published protocols [17, 18]. In brief, mice were anesthetized with ketamine/xylazine (80 mg/kg and 8 mg/kg, resp.), and the neck was incised to expose the left common carotid artery (LCCA). Two separate LCCA ligatures were made using 6.0 surgical sutures. The left internal carotid artery (LICA) and the left pterygopalatine artery were clipped. The LCCA was perforated with a 27-gauge needle, and a sterile 5.0-nylon monofilament with cautery-enlarged tip was inserted through the hole and pushed up to the clip in the LICA. Both microvascular clips were then removed. The filament was advanced and inserted about 10 mm into the middle cerebral artery. After 60 minutes of occlusion, a third ligature was placed around the LCCA, the filament was removed, and the LCCA was permanently ligated.

### 2.3. Treatment

Immediately after removal of the filament and wound closure with nylon suture, the mice were allocated to one of three groups: 0.3 mL (300 *μ*L) saline treatment (0.9% sterile NaCl solution, sham injection), single peritoneal injection of bevacizumab (Avastin, Genentech/Roche, 75 *μ*g/300 *μ*L), or single peritoneal injection of sildenafil (Revatio 0.8 mg/mL solution for injection, Pfizer Limited, Sandwich, United Kingdom; 24 *μ*g/300 *μ*L). An additional group of 7 mice received a single bevacizumab injection 6 h after induction. All mice were given fluid replenishment (0.5 mL sterile saline) postoperatively, and topical lidocaine gel was placed on the incision site for pain relief.

### 2.4. Triphenyltetrazolium Chloride Staining

At 24 hours after MCAO induction, the animals were euthanized with carbon dioxide. The brains were removed, incubated in 0.5% 2,3,5-triphenyltetrazolium chloride (TTC) solution in 0.9% saline for 10 minutes at 37°C, and cut coronally.

### 2.5. Fluorescent Gelatin Perfusion for Brain Vascular Imaging

Vascular imaging was performed by transcardial perfusion of gelatin containing fluorescently labeled bovine serum albumin (BSA), as previously reported [18, 19]. Briefly, the mice were terminally anesthetized with pentobarbital overdose, and a series of solutions was perfused transcardially as follows: 20 mL heparinized 0.9% saline, 10 mL 2% type A gelatin in saline with fluorescein-conjugated BSA (approximately 0.01 mg/mL), and 2 mL 4% type A gelatin in saline with cyanin-conjugated BSA. Thereafter, the ascending aorta was clamped with a hemostat while under pressure, and the animal was immersed in an ice-water bath to congeal the gelatin. Perfusion of both hemispheres (unaffected and injured) was compared.

### 2.6. Immunostaining

Cryosections of untreated (saline) MCAO induced brain sections were taken at 24 h, washed with phosphate buffer saline ×1, blocked with 2% BSA in phosphate buffer saline with 0.5% Triton X-100 for 15 min, and incubated at 4°C overnight with the primary antibody: rat anti-CD45 (1 : 100, Millipore, Temecula, CA). The sections were washed with 0.2% PBS with 0.5% Triton X-100 and incubated at room temperature for 1 h with the secondary antibody: goat anti-rat IgG Alexa Fluor 488 (1 : 200) (Molecular Probes, Invitrogen Corporation, Carlsbad, CA, USA). The brain sections underwent nuclear counterstaining with DAPI (Invitrogen). Images were generated using a conventional fluorescence microscope (Fluoview X; Olympus, Tokyo, Japan). Excitation wavelengths were 405 nm for DAPI and 488 nm for Alexa Fluor.

### 2.7. Calculation of Lesion Volume and Hemispheric Asymmetry

Digital images of the TTC-stained coronal sections were analyzed for lesion volume and edema using Fiji imaging processing software (ImageJ, National Institutes of Health, USA). Stroke area and penumbra and hemispheric volume were calculated. The stroke lesions were identified as white areas completely devoid of TTC staining, and the penumbra was segmented by any color noticeably lighter than healthy tissue, which appears bright red [20]. Total stroke area was defined as the sum of the stroke and penumbra region. Brain edema was defined by hemispheric asymmetry, calculated as the ratio of infarcted hemisphere to normal healthy hemisphere.

### 2.8. Molecular Analysis

#### 2.8.1. RNA Extraction

Brain tissues from 11 mice after MCAO induction, 6 treated 6 hours after induction by bevacizumab, were dissected and frozen in RNAlater (Life Science Division, Sigma-Aldrich, St. Louis, MO, USA). The middle third of each hemisphere was used. The mRNA samples were analyzed in pairs per mice (injured and uninjured hemisphere) and also pooled (normal unaffected hemispheres versus stroke hemispheres, for treated and untreated groups). Total RNA was isolated using TRIzol*™* reagent (Life Technologies, Invitrogen) according to the manufacturer's protocol and then reverse-transcribed into cDNA using random hexamers (Amersham Biosciences, Buckinghamshire, UK) and Moloney murine leukemia virus reverse transcriptase (Promega, Madison, WI, USA).

#### 2.8.2. Real-Time Quantitative PCR

Real-time quantitative PCR (RT-qPCR) was applied to study gene expression of apoptosis-related BAX and BCL-2 genes, inflammatory-related CD45 and MIP-2 (IL-8), and stress-related HO-1 genes in the brain using gene-specific primers (Table 1). The reaction efficiency was tested using a standard curve. Gene expression was normalized to mouse beta actin, a housekeeping gene. StepOne*™* Software v2.2.2 (Applied Biosystems, Foster City, CA, USA) was used for RT-qPCR. Reactions were performed in a 10 *μ*L volume containing 1 *μ*L cDNA, 0.5 *μ*M of each of the forward and reverse primers, and buffer included in the Master Mix (SYBR® Green I; Applied Biosystems).

Cycling conditions consisted of an initial denaturation step of 95°C for 10 min followed by 50 cycles of 1 min at 95°C and 1 min of annealing and extension at 60°C. Duplicate RT-qPCR reactions were performed for each gene to minimize individual tube variability, and an average was taken for each time point. Threshold cycle efficiency corrections were calculated, and melting curves were obtained using cDNA for each individual-gene PCR assay. The results were quantified by the comparative threshold cycle (Ct) method, where ΔΔCt = ΔCt (sample) − ΔCt (reference  gene) (Data Assist*™* Software v2.2.2, Applied Biosystems).

### 2.9. Statistical Analysis

Experimental data are expressed as mean ± standard error. Student's *t*-test was used to determine the significance of differences between groups. *p* < 0.05 was considered significant.

## 3. Results

**Figure 1:**
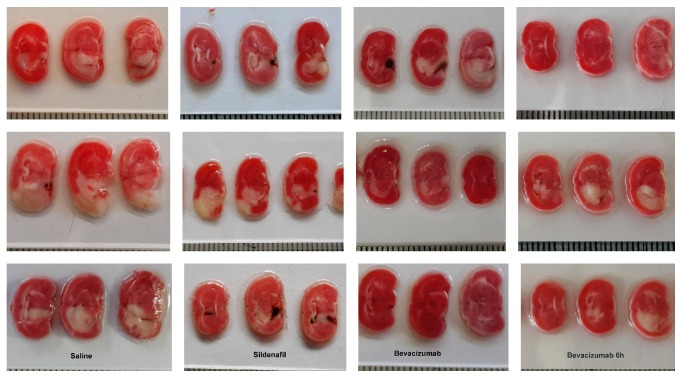
TTC (2,3,5-triphenyltetrazolium chloride) staining of MCAO infarcted brain tissue from mice treated with saline (control, first column), sildenafil (2nd column), and bevacizumab (3rd and 4th columns). Infarcted tissue is pale while healthy tissue is pink. It is evident that the mice treated with bevacizumab (immediate and late) have the largest amount of healthy tissue, indicating a neuroprotective effect. Each line represents different animal.

Mean values of the stroke and edema parameters after MCAO induction in the mice treated with saline, bevacizumab, or sildenafil are shown in Table 2 and Figure 1. The early bevacizumab-treated animals had a significantly greater mean penumbra area (salvageable tissue surrounding the infarct) than both the control mice (*p* = 0.03) and the sildenafil-treated mice (*p* = 0.003) and both groups of bevacizumab had smaller total stroke area than the other groups. The percentage of injured brain hemisphere relative to total healthy hemispheric tissue was lower in the bevacizumab-treated mice than in the sildenafil-treated mice (*p* = 0.34). The saline-treated animals had the lowest hemispheric asymmetry. There was no decrease in brain edema after ischemic injury in either the bevacizumab- or sildenafil-immediately treated group.

**Figure 2:**
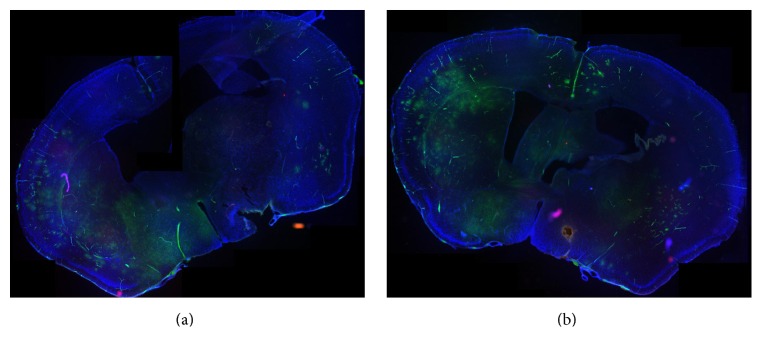
Perfusion studies of injured hemisphere versus control. Two brain sections following fluorescent perfusion of MCAO induced brains without treatment (saline group). Note the good vascularization on the healthy side, with infarct and lack of perfusion in the infarcted area. (Low magnification ×5, panoramic view).

**Figure 3:**
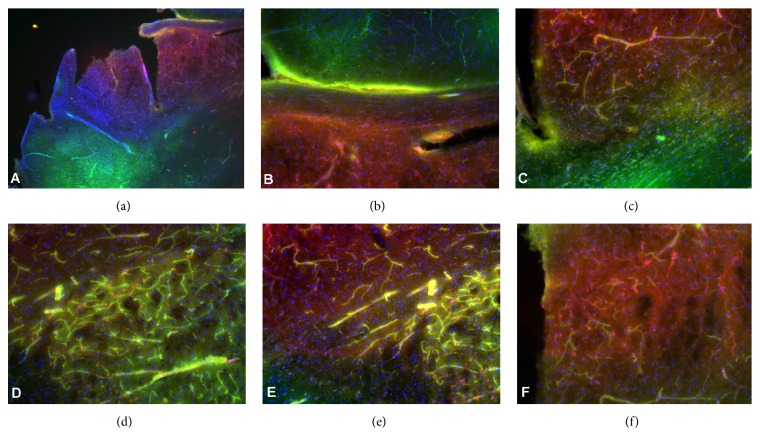
CD45 staining (red) of brains section of mice following MCAO induction without treatment (saline injected, controls) and perfusion with fluorescent gelatin (green). The nuclei are stained with DAPI (blue). (a) CD45 immunostaining (red) showing infiltration of the inflammatory cells to the injured hemisphere and increased cellularity (DAPI, blue) (×5), while the unaffected hemisphere is well perfused (green). (b) Higher magnification of the injured versus unaffected brain (×20). (c) Incomplete perfusion of the vessels in the infarct area, with extravasation of inflammatory cells to the tissue, as compared to the unaffected brain (green) without inflammatory cells. (d–f) Incomplete vascularization with intra- and extracellular inflammatory cells (×40).

**Figure 4:**
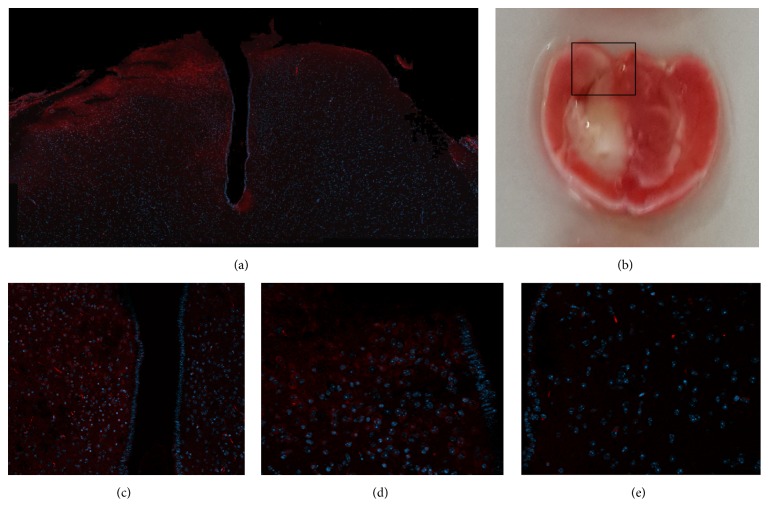
Immunohistochemistry staining for inflammatory cells using CD45 marker, demonstrating Inflammatory infiltration to the infarct area. (a) Inflammatory cells in the injured hemisphere but not in the other side (×20). (b) The location of the area immunostained in (a) is demonstrated in TTC staining (×5). (c) Higher magnification of (a) (×40). (d) The injured hemisphere with positive staining for inflammatory cells. (e) The unaffected hemisphere, negative for CD45 staining.

Figure 2 shows the lack of perfusion in the injured hemisphere as compared to the unaffected hemisphere of untreated mice (control). Immunostaining for inflammation (CD45) of the same brains (Figure 3) shows the extravascular infiltration of inflammatory cells into the damaged area, where the vascular perfusion was interrupted.  Figure 4 shows infiltration of CD45 into the stroke area, in the delayed bevacizumab-treated brain.

Table 3 shows the MCAO-related morbidity and mortality in the three groups. Mortality was categorized as either perioperative (traumatic bleeding during the procedure or anesthesia related) or postoperative (death after surgery). Morbidity was defined as visible neurological deficit after surgery and/or treatment. The highest mortality (75%) was detected in the late bevacizumab injection, followed by 60% in the saline group, probably due to three experiments where none of the mice survived the anesthesia. Following the MCAO induction, the bevacizumab-treated mice had lower morbidity and mortality rates than controls. The sildenafil-treated group had lower mortality than controls but higher morbidity.

The molecular analysis of the untreated group versus the bevacizumab 6 h treated group showed significantly increased BAX levels and BAX/BCL-2 ratio (5.07-fold versus 0.90-fold for BAX and BAX/Bcl-2 ratio 3.84 versus 0.51) and increased CD45 levels in the bevacizumab 6 h treated group (2.04-fold) versus untreated group (0.84-fold). SOD1 and GFAP levels remained at baseline in the untreated group and mildly reduced (0.82 fold, NS) in the 6 h bevacizumab-treated group; MIP-2 remained at baseline in both groups.

## 4. Discussion

This study shows that bevacizumab, a VEGF inhibitor, has a neuroprotective effect on mice after MCAO induction. The bevacizumab-treated mice had a lower volume of injured brain and a larger penumbra, meaning more salvageable tissue than the untreated mice. Furthermore, injection 6 h after MCAO induction still showed a neuroprotective effect with reduced brain edema. However, there was no between-group difference in brain edema in the early treated groups. We did not provide any evidence to support the assumption that bevacizumab decreases the leakage and therefore protects the brain. However, the neuroprotective effect might be exerted through the reduced inflammatory cell infiltration into the injured area. Inhibition of the inflammatory reaction plays a role in the neuroprotective effect. We hypothesize that this neuroprotective effect of bevacizumab is directly related to the anti-inflammatory properties of the drug. It is of note that the mortality was highest in the group treated only 6 hours after MCAO induction (Table 2). This warrants further investigation.

Molecular analysis showed a neuroprotective effect in reducing apoptosis reaction. While CD45 was elevated in the 6 h bevacizumab-treated group, the untreated group showed a trend to receding inflammatory reaction, which is not significant to discuss protection or destruction. MIP-2 and GFAP levels were similar near baseline in both groups. Previous studies from our laboratory have demonstrated protective effect in reducing acute inflammation in stroke model in TLR4 knockout mice in brain [20] and optic nerve [21].

We have demonstrated here the increased infiltration of inflammatory cells to the damage area in the brain (using immunohistochemistry) in untreated mice but showed increased molecular gene expression levels of CD45 but not MIP-2 in the 6 h treated brain. We assume that the CD45 represent inflammatory reaction that can be protective or destructive, depending on its expression levels. Further analysis of IL-8/MIP-2 and GFAP did not show differences between the groups.

It has been postulated that there might be a connection between hypoxia and inflammations [22]. This relationship could be explained by the fact that the hypoxic endothelium expresses inflammatory chemokine receptors, such as CXCR4 and CXCR7, as well as the angiogenic chemokines (such as CXCL8 and CXCL1) [22]. These are all regulated by the transcription factor hypoxia inducible factor and serve as potential future drug targets for anti-inflammatory drugs. We measured the inflammatory-related gene expression and confirmed the increased inflammatory effect after stroke induction. Immunostaining for CD45 and molecular expression levels confirmed its effect.

Unlike bevacizumab, sildenafil did not exert a significant generalized neuroprotective effect. Although sildenafil was found to reduce the inflammatory reaction in models of demyelinating brain injury [14], it was not effective in the MCAO model. There was no significant difference in poststroke brain edema between sildenafil- and saline-treated mice.

These findings contrast with earlier clinical studies. Bevacizumab has been shown to induce cerebral ischemic events in patients being treated for glioma and to reduce brain edema in patients with brain tumor [3, 7]. We attribute its protective effect to the restriction of proinflammatory activities. Sildenafil has been found to protect against cerebral edema [15, 16] and to serve as an appropriate therapy for patients with stroke [23]. Accordingly, in an experimental study of the poststroke effects of bevacizumab [24], researchers treated Sprague-Dawley rats with bevacizumab or saline following induction of MCAO and analyzed the findings with positron emission tomography in conjunction with behavioral analysis. Their results suggested that bevacizumab has no effect on functional recovery after stroke [24]. However, positron emission tomography showed a decreased metabolic demand for glucose and inhibition of vascular formation, which could explain our finding of a neuroprotective effect. In an analogous study of sildenafil, rats in the study group had consistently better behavioral scores than controls and a reduced cerebral infarction volume [25]. Transmission electron microscopy showed a significant decrease in the number of degenerated neurons in the sildenafil group, and molecular analysis showed a significant decrease in the extent of synapse damage via the cGMP-dependent Nogo-R pathway.

The difference between similar studies in the brain [24, 25] and our study has several explanations. These experimental studies used rat models of stroke instead of a mouse model which we utilized. A much smaller number of animals (*n* = 8–12) were used in these studies compared to our work (bevacizumab 19/51 and sildenafil 16/51) with much higher doses of the study drugs (up to 32 mg/kg ×3) [24, 25]. The time course of our experiments was also different. Our animals were investigated only 24 hours after MCAO induction, whereas other studies used varying time points [24, 25]. Also, the neuroprotective effect of bevacizumab was observed in the context of solid tumors, where it could be due to a decrease in glucose demand in neural tissue, as shown on positron emission tomography [24].

Sildenafil was also found to have a protective effect in various organ systems, such as heart and kidney [13], probably via a reduction in oxidative stress in ischemia reperfusion injury. Specifically, sildenafil induced mitochondrial biogenesis in the injured cells in the renal cortex. Mitochondrial dysfunction/disruption of mitochondrial biogenesis is one of the mechanisms underlying ischemia/reperfusion injury in the kidney and apparently plays a role in acute kidney injury and renal tubular dysfunction [13]. It has also been implicated in ischemia reperfusion injury in the brain. Sildenafil was found to induce mitochondrial biogenesis in the mouse renal cortex [12], and a postmortem analysis of brain tissue from patients after ischemic events showed that mitochondrial oxidative stress was responsible for neuronal cell death [23]. Therefore, sildenafil is considered a promising therapeutic agent for patients with acute kidney injury or stroke.

However, it must be mentioned that the fundamental physiology of neural tissue is different to other tissues, making it less forgiving to ischemic injury. It has been shown that mitochondrial dysfunction due to oxidative stress after ischemic injury is most likely the mechanism of neuronal damage in stroke [23]. It is likely, however, that sildenafil has a less protective effect in neural tissue compared to other tissues in the body due to the less forgiving nature of the central nervous system to ischemic injury.

## 5. Conclusions

This study demonstrated that bevacizumab has a slight neuroprotective effect after ischemic stroke, whereas sildenafil does not. This is in contradiction to previous studies [24, 25]. However, we have utilized a larger sample size. Caution should be used in attempting to identify neuroprotective drugs. Neural tissue is complex, and a large amount of animals/samples are needed before meaningful results can be obtained.

## Figures and Tables

**Table 1:** List of primers.

Gene	Forward primer sequence (5′-3′)	Reverse primer sequence (5′-3′)
Mouse ACTB	TAGGCACCAGGGTGTGATGGT	CATGTCGTCCCAGTTGGTAACA
Mouse SOD-1	GCCCGGCGGATGAAGA	CGTCCTTTCCAGCAGTCACA
Mouse HO-1	CAGGTGTCCAGAGAAGCTTT	TCTTCCAGGGCCGTGTAGAT
Mouse CD45	GAACATGCTGCCAATGG	TGTCCCACATGACTCCTT
Mouse BAX	CTGAGCTGACCTTGGA	GACTCCAGCCACAAAG
Mouse BCL-2	CCTGTGGATGACTGAG	GAGCAGGGTCTTCAGA
Mouse TNF-*α*	TCT CAA AAT TCG AGT GAC AAG C	ACT CCA GCT GCT CCT CCA C
Mouse GFAP	CCAGCTTCGAGCCAAGG	GAAGCTCCGCCTGGTAG
Mouse MIP-2	CAGGGTGTGATGGTGGGAAT	TGCTCTGGGCCTCGTCA

**Table 2:** Penumbra, stroke, and brain edema in sildenafil-, bevacizumab- (immediate and 6 h), and saline-treated MCAO induced mice.

Treatment	*N*	Mean penumbra area (mm^3^)	Mean stroke area (mm^3^)	Total stroke area (mm^3^)	Total injured hemisphere (mm^3^)	% of injured hemisphere	Normal hemisphere	Hemispheric asymmetry (ratio)
Sildenafil	16	5.7 ± 2.02	12.72 ± 6.76	18.42 ± 5.41	32.63 ± 1.60^*∗*^	56.10 ± 15.31	29.68 ± 3.13	1.099

Bevacizumab	19	7.3 ± 3.5	10.50 ± 5.53	17.79 ± 5.80	34.67 ± 2.04^*∗*^	51.0 ± 15.52	31.89 ± 3.25	1.086
	7	2.4 ± 2.0	7.40 ± 5.42	9.80 ± 8.00	32.90 ± 2.93	29.00 ± 22.3	33.51 ± 2.23	0.982

Saline	16	4.5 ± 2.03	14.70 ± 6.75	19.20 ± 6.38	32.72 ± 2.95	58.09 ± 16.7	30.70 ± 3.20	1.065

^*∗*^ *p* = 0.03 versus saline and *p* = 0.003 versus sildenafil.

**Table 3:** Mortality and morbidity in treated rats after MCAO induction.

	Saline	Bevacizumab		Sildenafil
	Imm.	Imm.	6 h	Imm.
*Total number of mice at study onset *	58	32	35	27
*Mortality *				
After surgery	15	5	14	1
After anesthesia	20	4	9	1
Total	35 (60%)	9 (28%)	23 (68%)	2 (0.74%)
*Morbidity*				
After surgery	3 (13%)	0	0	6 (24%)
Survived procedure	20	23	12	19
*Included in study*	*16*	*19*	*12* ^*∗*^	*16*
No stroke	4 (20%)	3 (13%)	0 (0%)	1 (6.3%)
Extreme damage	0	1 (4.3%)	0 (0%)	2 (10.5%)

Imm.: immediately injected after MCAO induction. ^*∗*^7 of 11 for histology (TTC) and immunohistochemistry and 5 for molecular analysis.
